# Supplementary material for: Natural allelic variation of FRO2 modulates Arabidopsis root growth under iron deficiency
Source: Nat Commun. 2017 May 24;8:15603. doi: 10.1038/ncomms15603 (PMC5458102; doi:10.1038/ncomms15603)
Supplement: Supplementary Information — Supplementary Figures, Supplementary Tables and Supplementary References [file ncomms15603-s1.pdf]

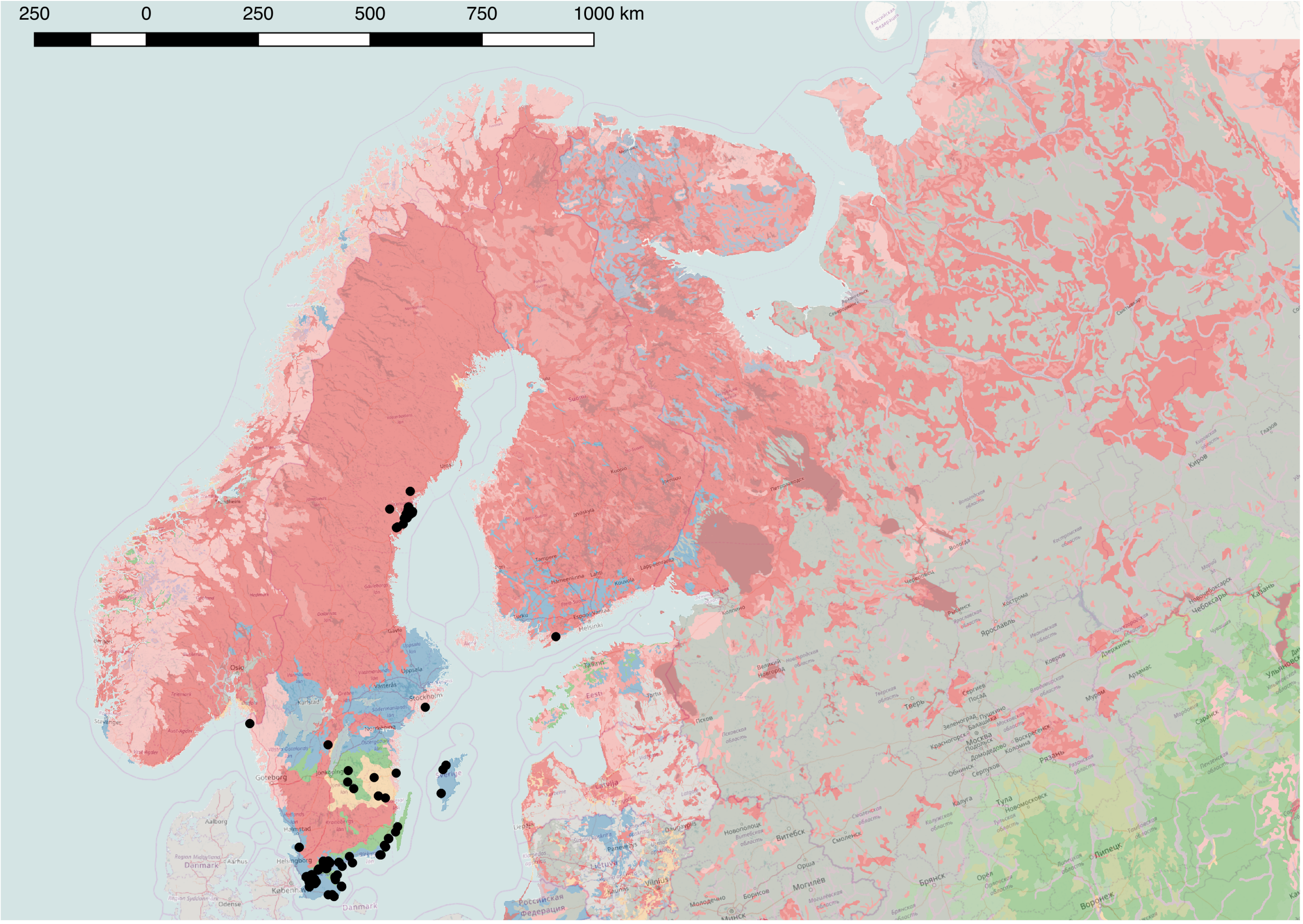

Supplementary Figure 1. Location of origin for the accessions used in this study. Each dot represents the sampling origin of one accession used for this study. The colors on the map indicate different soil types [1,2].

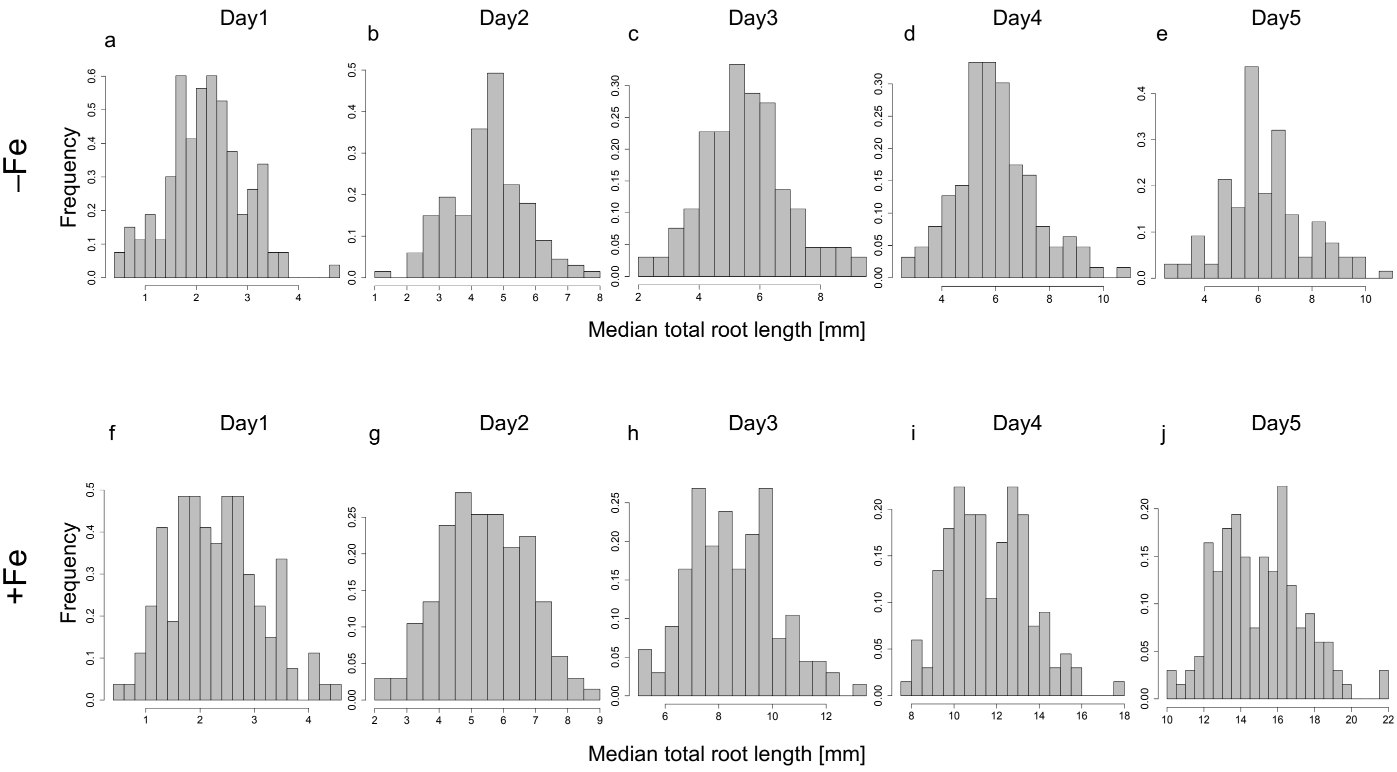

Supplementary Figure 2. Root length distribution of Swedish accessions. Histograms of median root lengths (mm) of accessions grown on Fe deficient (a-e) or Fe sufficient (f-j) growth conditions on each day of a 5 day time course. x-axis: median root length; y-axis: frequency.

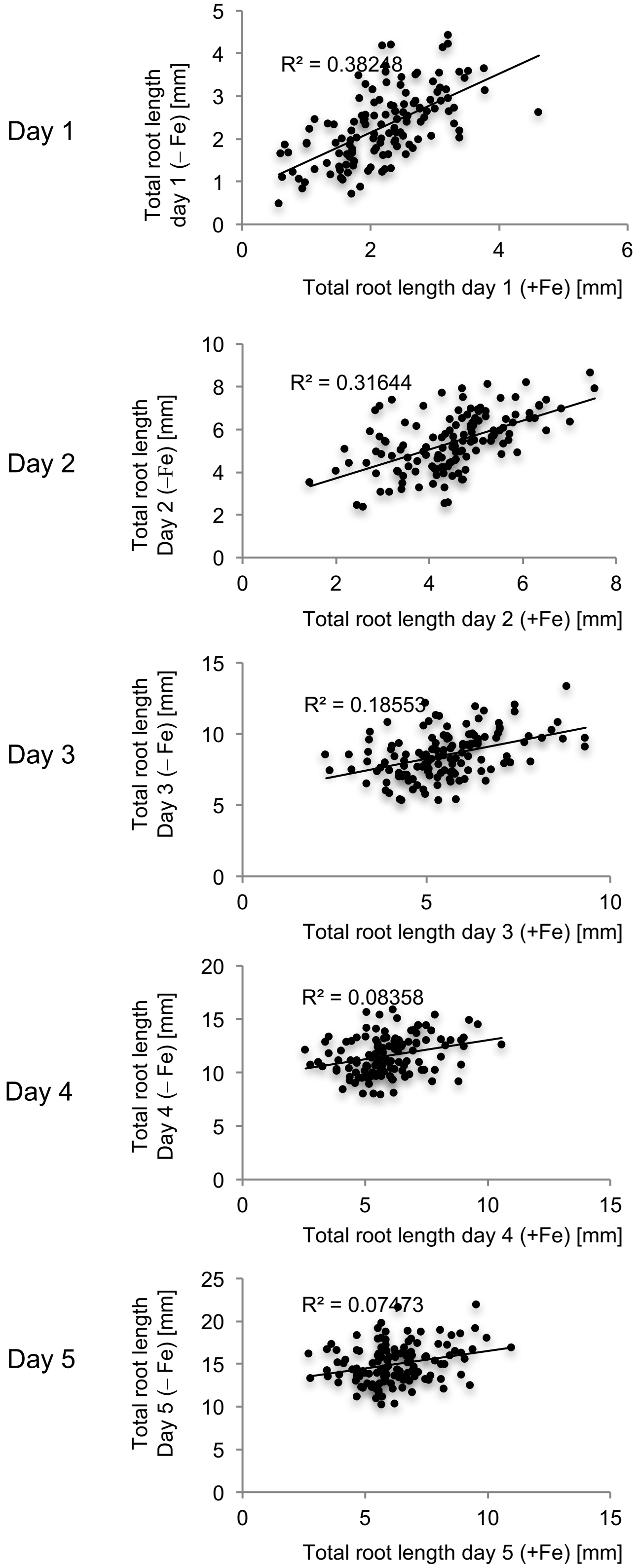

Supplementary Figure 3. Correlations for root length of Swedish accessions grown under +Fe and –Fe. Median root lengths (mm) of accessions grown in Fe sufficient (x-axis) or Fe deficient (y-axis) growth conditions on each day of a 5 day time course.

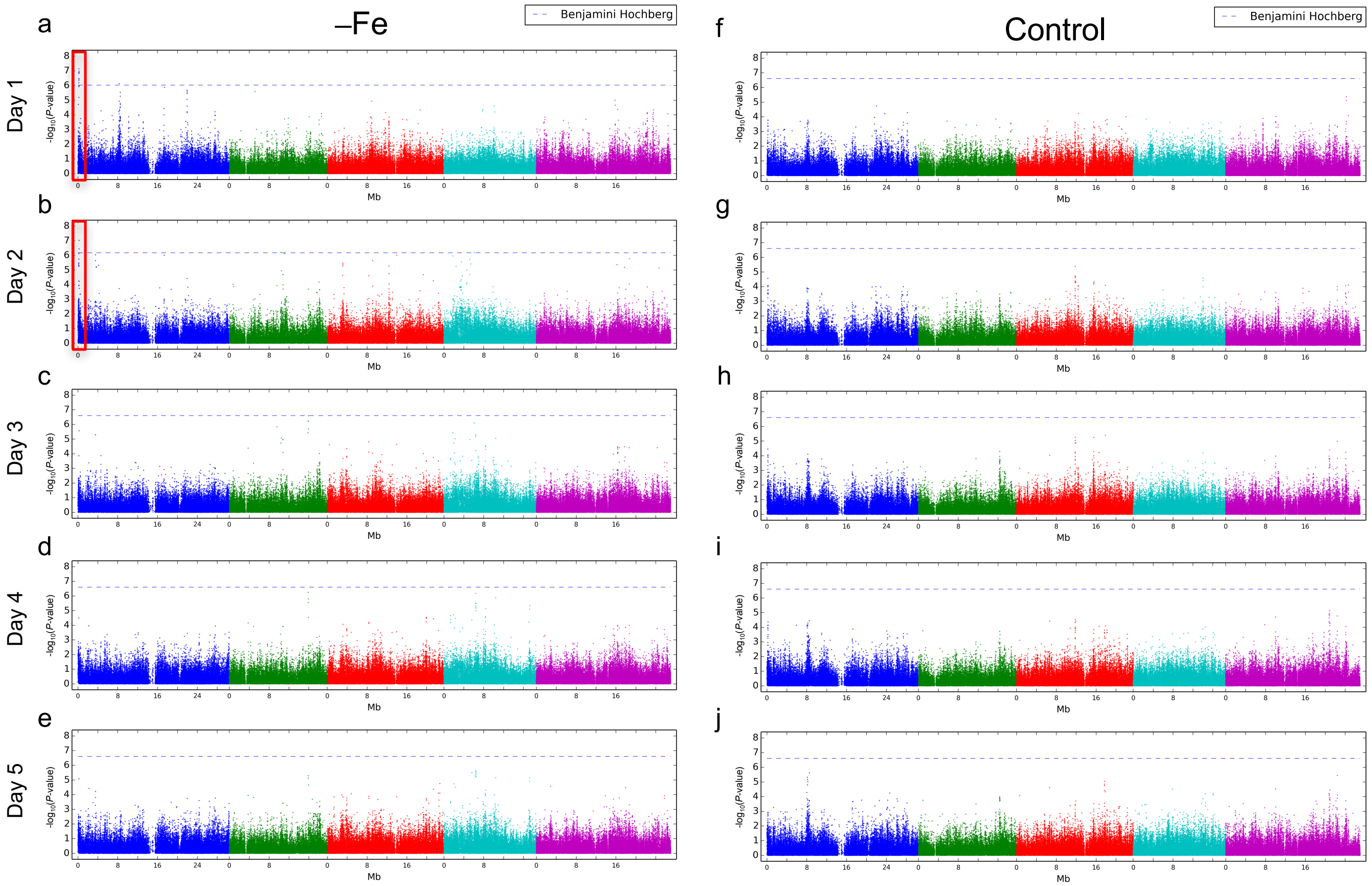

Supplementary Figure 4. GWAS for root length traits of Swedish accessions grown under +Fe and –Fe. Manhattan plots depicting genome wide SNP associations for median root length on Fe deficient (a-e) or Fe sufficient (f-j) growth conditions on each day of a 5 day time course. The chromosomes are depicted in different colors. The horizontal blue dash-dot line corresponds to a nominal 0.05 significance threshold after Benjamini Hochberg Correction. Red box indicates the significantly associated locus. x-axis: chromosomal position of SNP; x-axis: -$\log_{10}$(p-value).

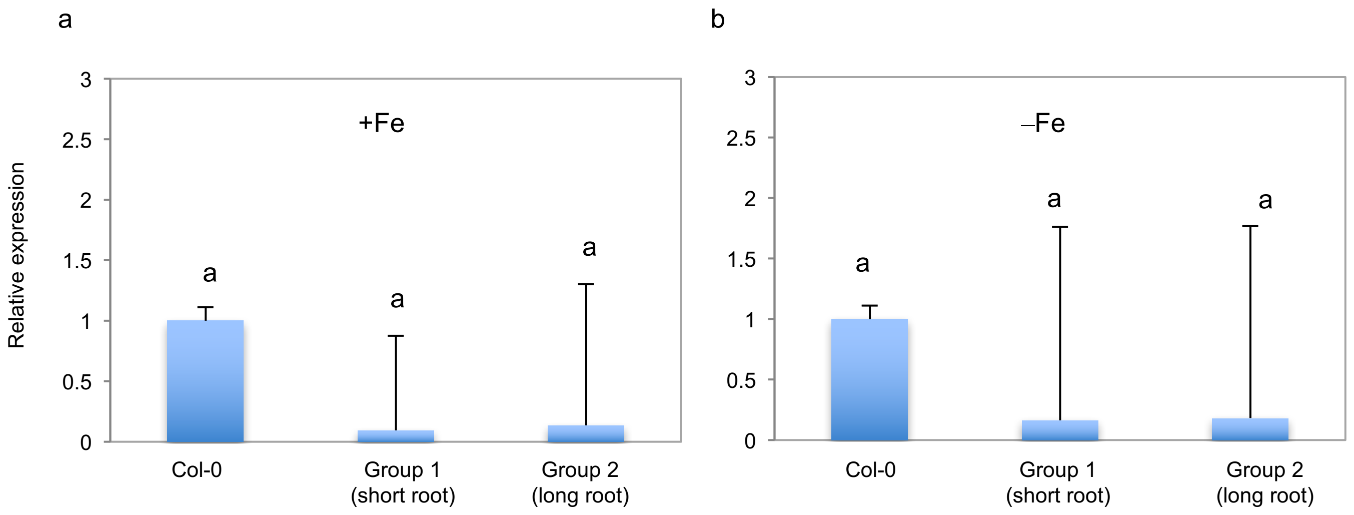

Supplementary Figure 5. ***FRO1*** **expression in extreme accessions under +Fe and –Fe.** *FRO1* expression in accessions displaying extreme root lengths (Group 1: Boo2-3, T1070, and Grön 14, short; and Group 2: TNY 04, TV-10, and TDr-16, long) as measured by qRT-PCR under +Fe (a) and under –Fe (b). Expression levels were normalized to expression in Col-0 under +Fe. Error bars: s.e.m. For qRT-PCR analyses, seeds were evenly placed on the mesh in a single row at a density of ~20 seeds/cm in two rows. Whole roots were sliced off and collected in liquid nitrogen. Around 50 plants were pooled together after 48 h of exposure to iron-deficient (–Fe) or iron-sufficient (+Fe) media.; data from three independent biological replicates each with two technical replicates are expressed as s.e.m. Letters indicate significantly different values at $p < 0.05$ determined by one-way ANOVA and Tukey HSD.

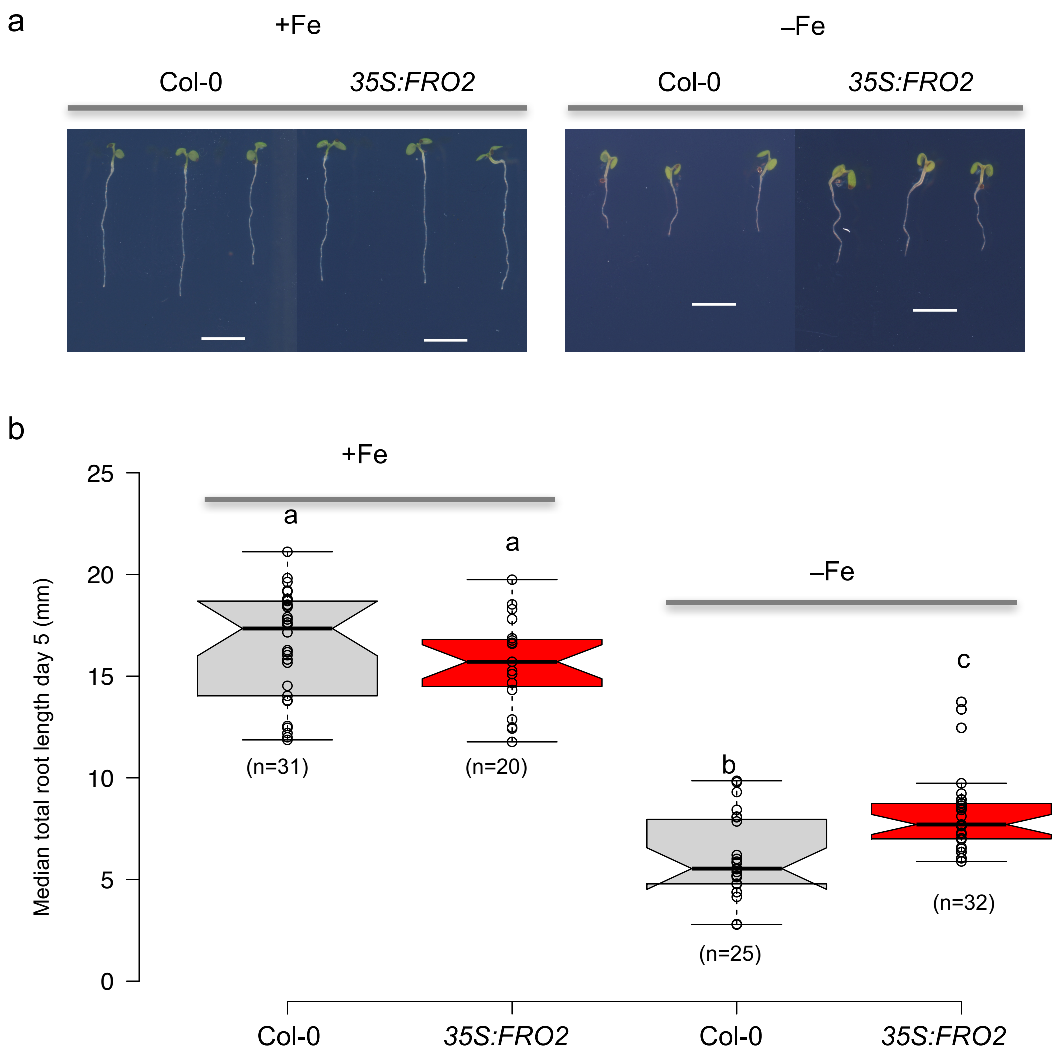

Supplementary Figure 6. *FRO2* regulates root growth under Fe deficiency.
(a) Representative seedlings of wild-type (Columbia Col-0) and *35S:FRO2* at 6 DAG grown on Fe sufficient (1X MS) or Fe-deficient medium (+300 µM FerroZine). Scale bars: 5 mm. (b) Boxplot of total root length of WT and *35S:FRO2* plants. Horizontal lines show the medians; box limits indicate the 25th and 75th percentiles; whiskers extend to 5th and 95th percentiles. Letters a, b, and c indicate significantly different values at $p < 0.05$ determined by one-way ANOVA and Tukey HSD.

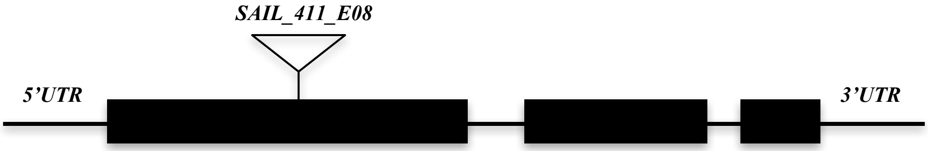

Supplementary Figure 7. Schematic representation of T-DNA insertion site in *AT1G01570.* Boxes indicate exons.

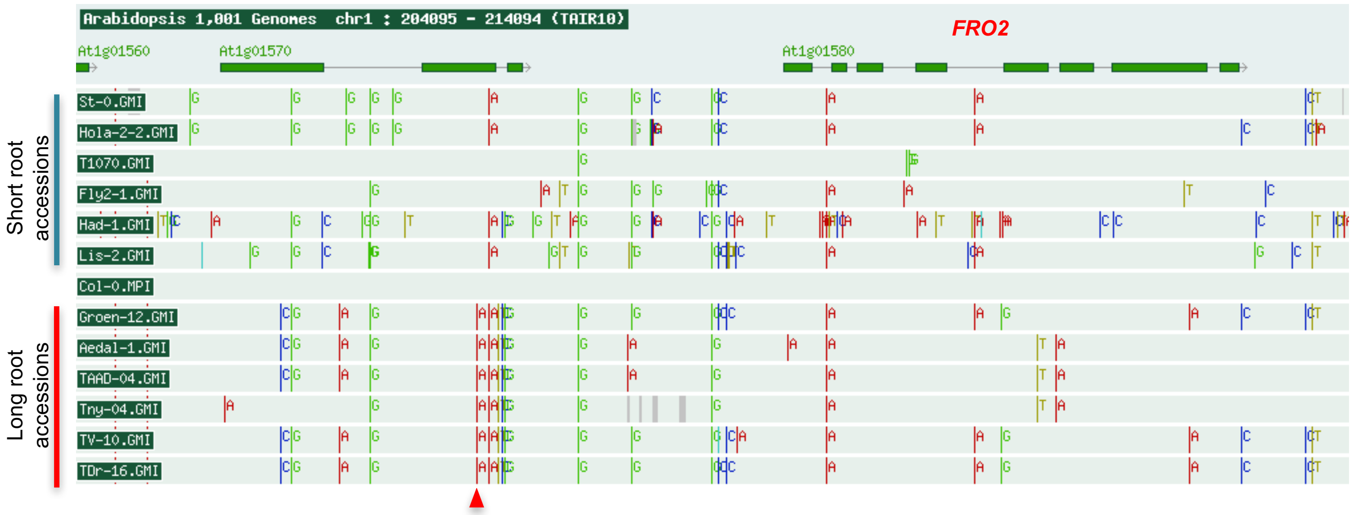

Supplementary Figure 8. SNPs around the *FRO2* locus in extreme accessions. Gene models and SNP polymorphisms among representative extreme accessions (6 accessions with short root phenotype and the reference allele of the marker SNP marked with blue line and 6 accessions with long root phenotype and the non-reference allele of the marker SNP marked with red line) for the genomic region surrounding the most significant GWA SNP. Col-0 (Reference accession) is shown in the middle. Red arrow: location of the significant GWA SNP. Only genomes that were available in the SALK 1001 genomes browser (http://signal.salk.edu/atg1001/3.0/gebrowser.php) as of May 2016 were considered.

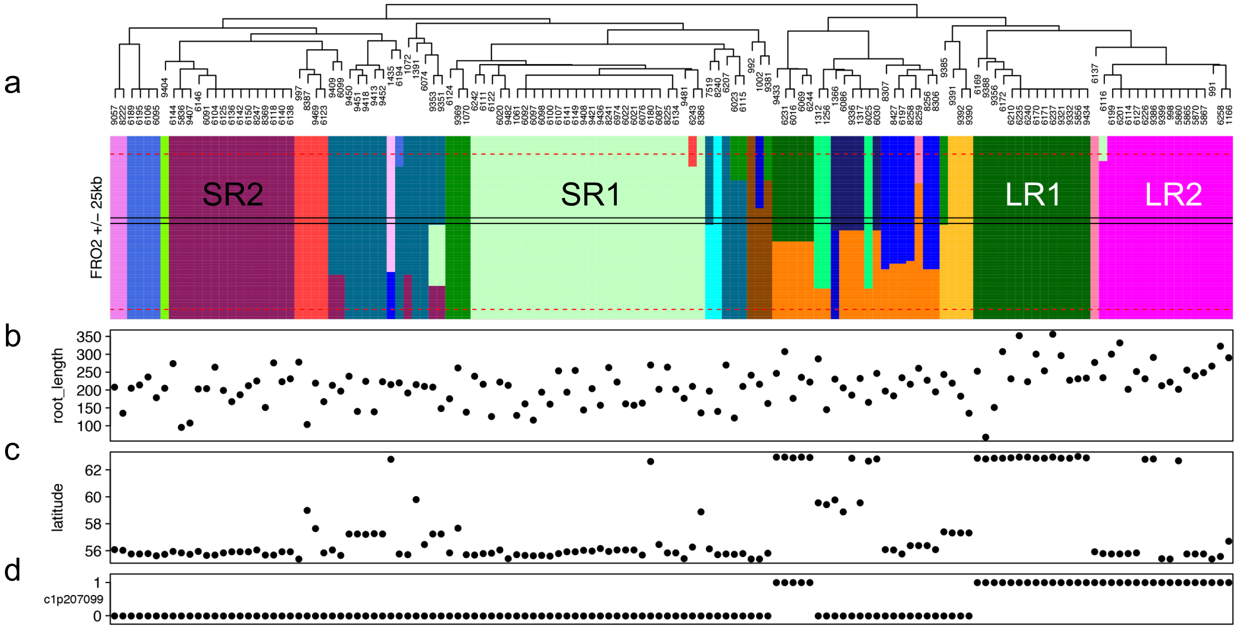

Supplementary Figure 9. Haplotype analysis around the *FRO2* locus. (a) The tree represents the genetic distance between accessions computed using SNPs 25 kb up and downstream of *FRO2*. X-axis: Accessions, Y-axis: SNPs. The horizontal black lines indicate the *FRO2* coordinates. Accessions that share the same color block share the same haplotype. (b) median root length of the accessions on day 2 (c) latitude of origin of the accessions (d) genotype of the accession at SNP c1p207099 (top GWAS hit).

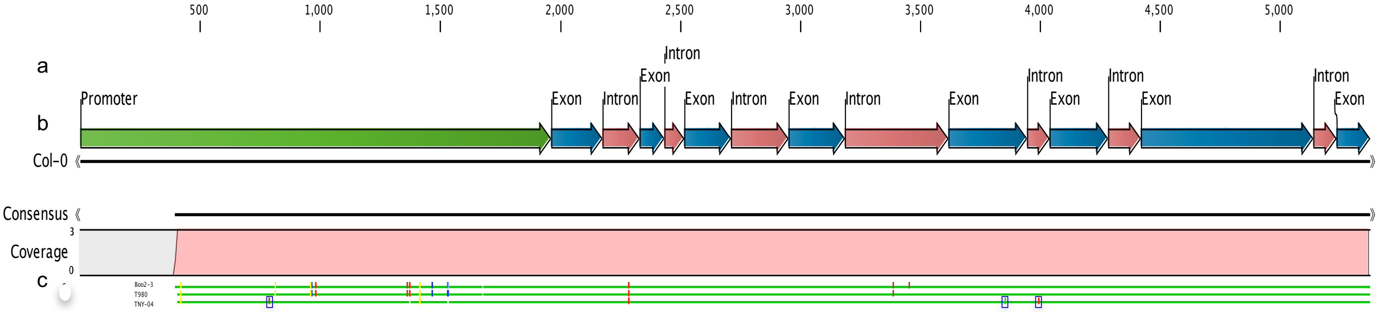

Supplementary Figure 10. SNP polymorphisms in regulatory and coding regions of *FRO2* gene as confirmed by Sanger sequencing. (a) Gene model showing promoter region, exons, and introns of *FRO2* are depicted in green, blue, and red respectively. *FRO2* genomic DNA sequences from Boo2-3, T980, and TNY 04 accessions were obtained by Sanger sequencing and aligned to Col-0 sequence using CLC Genomics Workbench. (b) Sequencing coverage (100%) is shown in faint red color. (c) SNP changes (compared to Col-0 reference) from extreme accessions are shown in colored vertical lines. A, T, G, and C nucleotides are represented as red, green, yellow, and blue lines respectively. TNY 04 *FRO2* specific polymorphisms (A in the regulatory region, T in the 5th exon and A in the 5th intron) are shown in blue boxes.

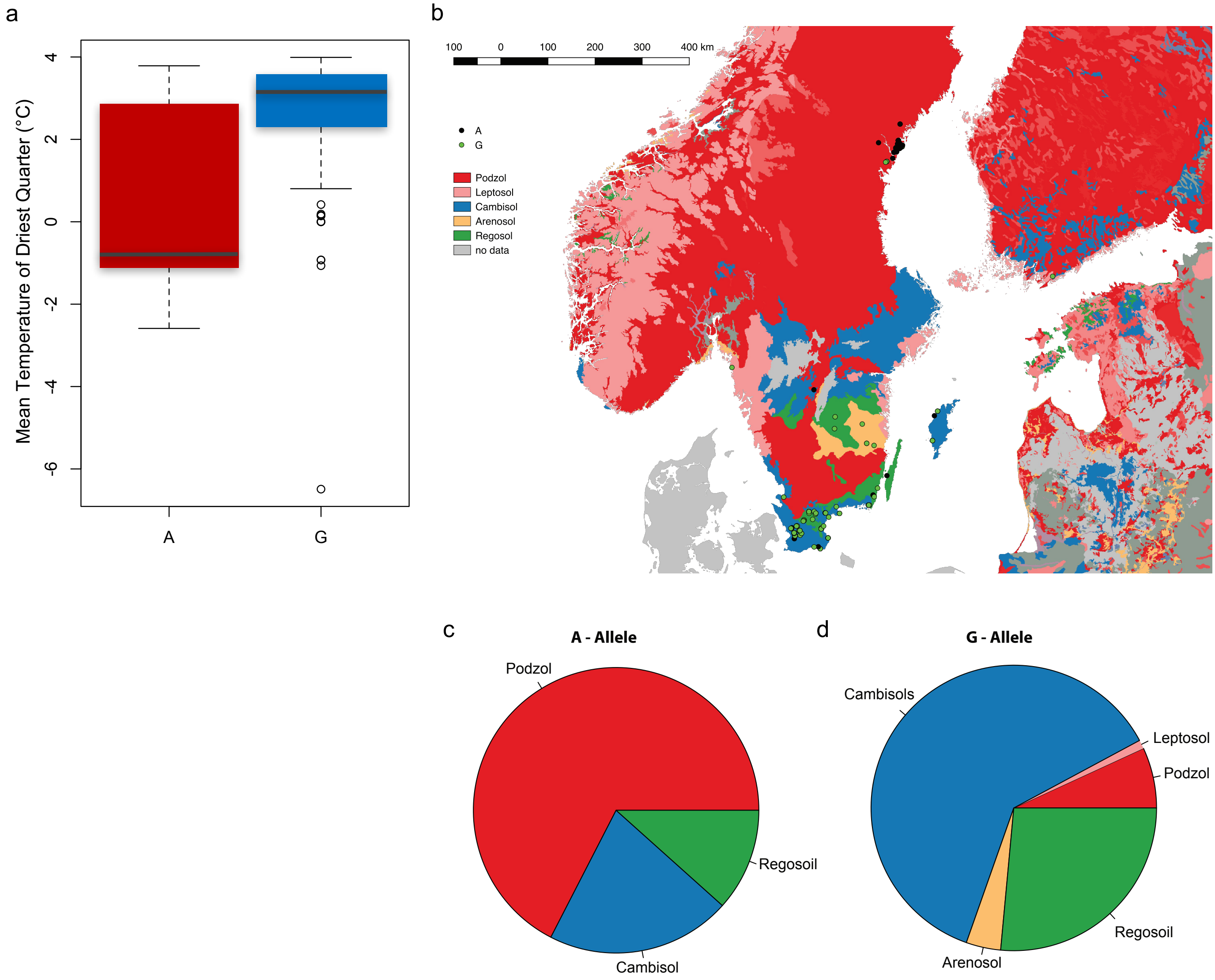

Supplementary Figure 11. Swedish *FRO2* alleles and soil types. (a) Box plot showing association between temperature (BIO9 = Mean Temperature of Driest Quarter) and top GWA SNP accessions. X-axis: SNP-allele; y axis: Mean Temperature of Driest Quarter (°C); whiskers, ± 1.5 times the interquartile range. (b) Geographic distribution of *FRO2* alleles. Each dot represents the sampling origin of one accession used for this study. Black dot: Accession that contains an A-allele of the marker SNP for *FRO2* that is associated with long roots on –Fe. Green dot: Accession that contains a G-allele of the marker SNP for *FRO2* that is associated with short roots on –Fe. The colors on the map indicate different soil types[1, 2]. (c, d) Pie charts showing the fraction of soil classes in the total sampling area of the minor allele (A-SNP) (c) and the major allele (G-SNP) (d).

Supplementary Table 1. Correlation analyses between SNPs and climate variables. A subset of the climate data used for analysis was obtained from Hancock et al. (2011)[3]. Raw data of climate variables (19 climate, latitude and longitude) were downloaded from the WorldClim project (www.worldclim.org)[4]. A linear model was used to test correlations between each of the variables and the root length phenotype. P-values were corrected for multiple testing.

Climate variable	p-value	adj. p-values
Mean Temperature of Warmest Quarter	9.27E-05	0.001947456
Annual Mean Temperature	0.001056184	0.022179873
Mean Temperature of Driest Quarter	0.001208186	0.025371896
Min Temperature of Coldest Month	0.00182659	0.03835839
Mean Temperature of Coldest Quarter	0.001855082	0.038956721
Temperature Annual Range	0.004797275	0.100742774
Temperature Seasonality	0.004836384	0.101564073
Latitude	0.008172001	0.171612014
Mean Diurnal Range	0.01003419	0.210717996
Longitude	0.019068509	0.400438684
Max Temperature of Warmest Month	0.070425309	1
Precipitation of Wettest Month	0.086479418	1
Isothermality	0.088563453	1
Precipitation of Driest Month	0.14462243	1
Precipitation of Wettest Quarter	0.226711396	1
Precipitation of Warmest Quarter	0.328325389	1
Mean Temperature of Wettest Quarter	0.368153327	1
Precipitation Seasonality	0.417305133	1
Precipitation of Driest Quarter	0.468015772	1
Annual Precipitation	0.480485261	1
Precipitation of Coldest Quarter	0.989213032	1

Supplementary Table 2. Correlation analyses between SNPs and climate variables. A subset of the climate data used for analysis was obtained from Hancock et al. (2011)[3]. Raw data of climate variables (19 climate, latitude and longitude) were downloaded from the WorldClim project (www.worldclim.org)[4]. A linear model was used to test correlations between each of the variables and the root length phenotype. P-values were corrected for multiple testing. To correct for population structure, we added to the linear model the first two eigenvectors that were obtained by performing a PCA on the SNP matrix that was used to run GWAS. PCA analyses were conducted with the eigenstrat() function from the AssocTests package in the R programming environment[5].

Climate variable	p-value	adj. p-values
**Max Temperature of Warmest Month**	0.01149964	0.241492436
**Mean Temperature of Warmest Quarter**	0.049057521	1
**Latitude**	0.251318849	1
**Precipitation Seasonality**	0.272428056	1
**Precipitation of Warmest Quarter**	0.348141157	1
**Temperature Seasonality**	0.393039641	1
**Precipitation of Driest Month**	0.39738128	1
**Longitude**	0.404103907	1
**Mean Temperature of Wettest Quarter**	0.428849271	1
**Annual Precipitation**	0.4770454	1
**Precipitation of Driest Quarter**	0.477137563	1
**Precipitation of Coldest Quarter**	0.482376127	1
**Temperature Annual Range**	0.485413855	1
**Precipitation of Wettest Month**	0.512771087	1
**Precipitation of Wettest Quarter**	0.553365978	1
**Mean Diurnal Range**	0.574678505	1
**Annual Mean Temperature**	0.734362061	1
**Isothermality**	0.772282861	1
**Mean Temperature of Driest Quarter**	0.863533801	1
**Mean Temperature of Coldest Quarter**	0.917948535	1
**Min Temperature of Coldest Month**	0.972172971	1

Supplementary Table 3. List of primers used in this study.

a) Primers used for genotyping of T-DNA insertion line.

Line name	Forward (5'to3')	Reverse (5'to3')
**SAIL_411_E08**	AATAATGTGAATGCACGGAGG	GACTACGTGAAGCTCTGGTGG

Left T-DNA border primer was LB2 (http://signal.salk.edu/tdnaprimers.2.html).

b) Primers used for cloning.

Gene	Forward (5'to3')	Reverse (5'to3')
FRO2	ATCGATAAGCTTGATATCAATC GTATTTTTGAAATTTCGAGC	CTGCAGGAATTCGATATCTCACC AGCTGAAACTGATAGATTC

c) Primers used for qRT-PCR.

Gene	Forward (5'to3')	Reverse (5'to3')
At1G01560	TCCTGAATGCAAACTGTGACCT	CCAAATGTCGATAGCTGCAGTG
FRO2	GCCACATCTGCGTATCAAGTT	TCCCAAACAAGCTACGACCA
AT1G01570	TGCAAGATCGTTGTATTCAGAGA	GGCCCAGAAGTTTCCCGTATAA
FRO1	ACATGTTCCCGATGATTCTACT	CCAAACTTGGCTCTCTTCTTC

Supplementary references

1. Panagos P. The European soil database. *GEO: connexion* **5 (7)**, 32-33 (2006).

2. Marc Van Liedekerke AJ, Panos Panagos. ESDBv2 Raster Library - a set of rasters derived from the European Soil Database distribution v2.0. The European Commission and the European Soil Bureau Network, CD-ROM, EUR 19945 EN (2006).

3. Hancock AM*, et al.* Adaptation to climate across the Arabidopsis thaliana genome. *Science* **334**, 83-86 (2011).

4. Hijmans RJ, Cameron SE, Parra JL, Jones PG, Jarvis A. Very high resolution interpolated climate surfaces for global land areas. *Int J Climatol* **25**, 1965-1978 (2005).

5. Team RC. R: A language and environment for statistical computing. R Foundation for Statistical Computing, Vienna, Austria. 2013. (2014).
